# Study of the Algorithm of Backtracking Decoupling and Adaptive Extended Kalman Filter Based on the Quaternion Expanded to the State Variable for Underwater Glider Navigation

**DOI:** 10.3390/s141223041

**Published:** 2014-12-03

**Authors:** Haoqian Huang, Xiyuan Chen, Zhikai Zhou, Yuan Xu, Caiping Lv

**Affiliations:** Key Laboratory of Micro-Inertial Instrument and Advanced Navigation Technology, Ministry of Education, School of Instrument Science and Engineering, Southeast University, Nanjing 210096, China; E-Mails: qhbhhq@163.com (H.H.); yyxy13406@gmail.com (Z.Z.); xy_abric@126.com (Y.X.); caipinglv@126.com (C.L.)

**Keywords:** underwater glider, inertial navigation system (INS), backtracking decoupling, adaptive extended Kalman filter (AEKF), quaternion expanded to the state variable

## Abstract

High accuracy attitude and position determination is very important for underwater gliders. The cross-coupling among three attitude angles (heading angle, pitch angle and roll angle) becomes more serious when pitch or roll motion occurs. This cross-coupling makes attitude angles inaccurate or even erroneous. Therefore, the high accuracy attitude and position determination becomes a difficult problem for a practical underwater glider. To solve this problem, this paper proposes backing decoupling and adaptive extended Kalman filter (EKF) based on the quaternion expanded to the state variable (BD-AEKF). The backtracking decoupling can eliminate effectively the cross-coupling among the three attitudes when pitch or roll motion occurs. After decoupling, the adaptive extended Kalman filter (AEKF) based on quaternion expanded to the state variable further smoothes the filtering output to improve the accuracy and stability of attitude and position determination. In order to evaluate the performance of the proposed BD-AEKF method, the pitch and roll motion are simulated and the proposed method performance is analyzed and compared with the traditional method. Simulation results demonstrate the proposed BD-AEKF performs better. Furthermore, for further verification, a new underwater navigation system is designed, and the three-axis non-magnetic turn table experiments and the vehicle experiments are done. The results show that the proposed BD-AEKF is effective in eliminating cross-coupling and reducing the errors compared with the conventional method.

## 1. Introduction

Underwater gliders play an important role and have become a mainstay in ocean missions, such as mine countermeasures, observation, survey, inspection and so on [1]. Underwater gliders are capable of performing long range missions with low energy consumption, low cost, and great endurance [2].

Their design must as simple as possible and the sensor quantity as few as strictly needed for the navigation system applied to an underwater glider because of the glider characteristics. The inertial navigation system (INS) is chosen as a better choice when GPS is unavailable underwater. The determination of navigation information is mainly dependent on the INS. The cross-coupling among three attitude angles (heading angle, pitch angle and roll angle) becomes more serious when pitch or roll motion occurs due to the misalignment errors between installation axis and the corresponding reference axis in the reference frame for the inertial measurement unit. This cross-coupling can make the determination of navigation information inaccurate or even erroneous. The pitch motion and roll motion are common for an underwater glider. There are two methods to solve the problem above: (1) establish and analyze the model of the inertial unit, and reduce and compensate the inherent error from the inertial system itself; (2) set up a model of the cross-coupling among the three attitude angles when pitch or roll motion occurs and derive the cross-coupling term, then eliminate the cross-coupling among attitudes and further smooth the filtering output to achieve improved accuracy and stability for the attitude and position determination. This paper focuses on the second method.

Li *et al.* [3] proposed an inverse Nyquist array (INA)-based method to design a precompensation matrix for approximate attitude decoupling. Hung *et al.* [4] proposed an adaptive neural network sliding-mode controller design approach with a decoupled method for nonlinear systems. In [5], a fuzzy decoupling method is proposed to solve the coupling problem. If it is hard to identify the system model, this intelligent decoupling solution is used. However, it is poor in engineering application because it needs a large amount of data, large computational resources and repetitive tests. References [6,7] proposed the differential geometry decoupling method, and the dynamic inverse decoupling method, respectively. A dynamic decoupling and static compensation procedure is designed to eliminate the cross-axis angular velocity coupling by Fang *et al.* [8].

After decoupling of attitude angles, the filtering method plays a very significant role in the process of attitude and position determination, to achieve high accuracy determination with high efficiency [9–11]. The Kalman filter (KF) is one of the most common examples for filtering. It can achieve the optimal estimation of states in a multi-input multi-output (MIMO) system under the conditions that prior knowledge about standard data deviation, the stochastic model of the transducer error, and the dynamic model of the system error are exactly known. Thus, the KF has been widely applied in vehicle attitude and position determination [12]. However, because of the system noises, measurements can be corrupted by white noise and the state estimation is approached with the minimization of the covariance of the estimation error, the KF is not suitable for nonlinear systems [13–17]. All kinds of algorithms are proposed to solve the problems of KF mentioned above.

Through the first-order linearization of the nonlinear system, extended Kalman filter (EKF) is able to achieve nonlinear estimation [18–21], but the state distribution is assumed as a Gaussian random variable (GRV). Large errors can be introduced in the true covariance of the transformed GRV and posterior mean. It makes EKF no longer effective in several special applications and sometimes even lead to divergence of the filter [22]. Moreover, the system with GRV is often unavailable in practice [23].

In the view of the above problems, this paper proposes the backing decoupling and adaptive extended Kalman filter (BD-AEKF) based on the quaternion expanded to the state variable method: (1) a backtracking decoupling method is proposed after establishing the model of the cross-coupling among three attitude angles and analyzing the reason for cross-coupling, to eliminate the cross-coupling during pitch or roll motion; (2) after decoupling, a state augmentation technique is applied in the process model and a specific measurement model is formulated, and adaptive extended Kalman filter (AEKF) based on quaternion expanded to the state variable is developed to further smooth the filtering output, therefore the accuracy and stability of attitude and position determination are improved greatly.

This paper is organized as follows: the dead reckoning model is presented in Section 2. In Section 3, cross-coupling models among attitudes when pitch or roll motion occurs are discussed in detail and we propose the backtracking decoupling method to solve the cross-coupling among three attitude angles. In Section 4, the AEKF based on quaternion expanded to the state variable is proposed to further improve the accuracy and stability of attitude and position determination. Simulations and analyses can be presented in Section 5. Results and discussion of experimental verification are presented in Section 6, followed by the overall conclusions presented in Section 7.

## 2. Dead Reckoning

**Figure 1.**
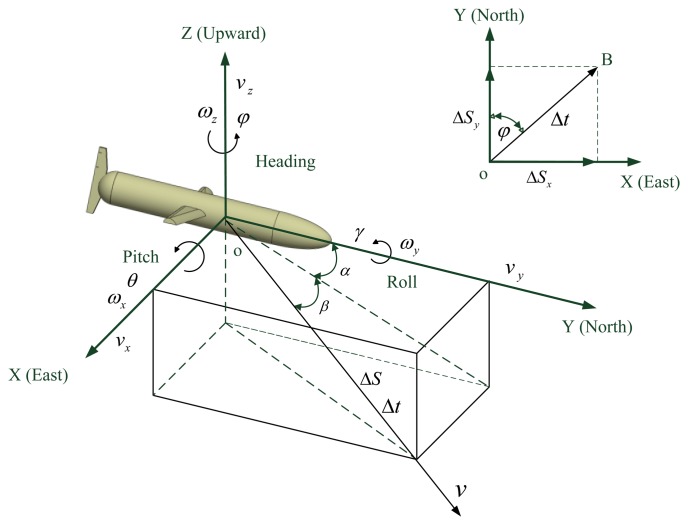
The underwater glider model.

When the glider glides at certain depth in the sea, the underwater circumstances are comparatively stable. The glider usually follows a sawtooth motion pattern in the vertical plane. The change of depth is measured by a depthometer or other instruments, and it is not the focus in this paper. The acquisition of navigation information mainly by the inertial system in the horizontal plane is studied in the content that follows. The sea current average velocity is approximately regarded as the constant at a certain depth and the glider glides with the sea current. Hence, the average velocity of glider is also considered as a constant. A model of an underwater glider is shown in Figure 1.

Assume that the glider has glided through the distance Δ*S* and the time Δ*t*. The velocity of the glider is estimated by Equation (1):

(1) $$v_{\text{dr}}=v_{\text{cur}}+v_{\text{noi}}$$

where *v_dr_* is the estimated velocity, *v_cur_* is the average velocity of the sea current, *v_noi_* is the additional noise;

The distance Δ*S* is estimated as follows:

(2) $$\Delta S=v_{dr}\Delta t$$

The position calculated approximately by DR is:

(3) $$\Delta S_{x}=\Delta S\text{cos}\theta_{i}\text{sin}\varphi_{i}$$

(4) $$\Delta S_{y}=\Delta S\text{cos}\theta_{i}\text{cos}\varphi_{i}$$

(5) $$\Delta S_{z}=\Delta S\text{sin}\theta_{i}$$

where *φ_i_*, *θ_i_* represent heading angle and pitch angle at the moment of *i*.

## 3. The Coupling Model and Backtracking Decoupling Method

### 3.1. The Cross-Coupling Model

It is essential to establish the cross-coupling model of attitude angles and analyze the reasons for cross-coupling. This section analyzes in detail the cross-coupling among three attitude angles when roll motion occurs. When pitch motion occurs, the model analysis and decoupling process are similar to the case of roll motion mentioned above.

As shown in Figure 1, the translational velocity of the underwater glider is defined as **v** = [*v_x_ v_y_ v_z_*]*^T^* and the angular velocity of the underwater glider is defined as **w** = [*ω_x_ ω_y_ ω_z_*]*^T^*, and attitude **η** = [*ψ θ γ*]*^T^* consists of heading angle, pitch angle and roll angle [2]. The equations of motion are [24]:

(6) $$\left[\begin{array}{ccc} M & D^{T} & 0_{3}\\ D & J & 0_{3}\\ 0_{3} & 0_{3} & I_{3} \end{array}\right]\;\left[\begin{array}{c} \dot{\text{v}}\\ \dot{\text{w}}\\ \dot{\eta } \end{array}\right]=\left[\begin{array}{c} (Mv+D^{T}w)\times w+F\\ (Dv+Jw)\times w+(Mv+D^{T}w)\times v+M\\ R(\eta )w \end{array}\right]$$

where $R(\eta )=[\begin{array}{ccc} 1 & \sin\gamma \tan\theta & \cos\gamma \tan\theta \\ 0 & \cos\gamma & -\sin\gamma \\ 0 & \frac{\sin\gamma }{\cos\theta } & \frac{\cos\gamma }{\cos\theta } \end{array}].$

**0**_3_ denotes matrices with all zero entries; **I**_3_ denotes identify matrix. Suppose the glider's *xy* and *yz* planes are symmetrical [25], which means that a diagonal matrix **M** contains mass terms *m_y_* and *m_x_* = *m_z_* including added mass. The diagonal matrix **J** also contains inertial terms *J_y_* and *J*_x_ = *J_z_* including added inertia:

(7) $$\begin{array}{ccc} M=\left[\begin{array}{ccc} m_{x} & 0 & 0\\ 0 & m_{y} & 0\\ 0 & 0 & m_{z} \end{array}\right] & \text{and} & J=\left[\begin{array}{ccc} J_{x} & 0 & 0\\ 0 & J_{y} & 0\\ 0 & 0 & J_{z} \end{array}\right] \end{array}$$

The cross-coupling matrix **D** is:

(8) $$D=\left[\begin{array}{ccc} 0 & mz_{cg} & mx_{cg}\\ -mz_{cg} & 0 & -my_{cg}-Z_{\dot{q}}\\ -mx_{cg} & my_{cg}+Z_{\dot{q}} & 0 \end{array}\right]$$

The center of gravity is located at [*x_cg_ y_cg_ z_cg_*]*^T^* and the dry mass is *m*. The cross added mass term *Z_q̇_* is nonzero if the glider is not symmetric about the *xz* plane. Because of overall buoyancy, hydrodynamic lift, and weight forces, **F** = [*F_x_ F_y_ F_z_*]*^T^* denotes external forces and **M** = [*M_x_ M_y_ M_z_*]*^T^* denotes external moments. **δ** = [*δ_e_ δ_r_*]*^T^* is the vector of fin deflections. The attack angle *α* and the slip angle *β* are defined as [2,26]:

(9) $$\alpha =\tan^{-1}\left(v_{z}/v_{y}\right)\;\text{and}\;\beta =\sin^{-1}\left(v_{x}/v\right)$$

with $v=\left|v\right|=\sqrt{v_{x}^{2}+v_{y}^{2}+v_{z}^{2}}$.

The sea circumstances are relatively stable at a certain depth, so the sea current average velocity could be regard as constant. Because the glider gliders with the sea current, the horizontal average velocity of glider is also thought as unchanged. Moreover, the roll angle *γ*(*t*) and roll rate *ω_y_*(*t*) are time-varying parameters. The remaining state vector becomes Equation (10) after transforming the linear velocities *v_x_*, *v_z_* into *α* and *β* using Equation (9):

(10) $$\chi :={\left[\begin{array}{cc} \chi_{\theta }^{T} & \chi_{\varphi }^{T} \end{array}\right]}^{T}$$

where:

(11) $$\chi_{\theta }:=T_{1}\;{\left[\begin{array}{ccc} \alpha & w_{x} & \theta \end{array}\right]}^{T}\;\text{and}\;\chi_{\varphi }:=T_{2}\;{\left[\begin{array}{ccc} -\beta & w_{z} & \varphi \end{array}\right]}^{T}$$

T_1_, T_2_ are the coefficients, respectively. The symmetry in the model is derived by negating *β* in Equation (10), and then the motion equations become as follows:

(12) $$E\dot{\chi }=f\left(\chi ,\delta ,t\right)$$

Make Equation (12) approximately linearized around the operation point *χ*_0_=0 and *δ*_0_=0 for nonzero roll motion *γ*(*t*) ≠ 0 and *w_y_*(*t*) ≠ 0, then:

(13) $$E\dot{\chi }=A_{\gamma }(t)\chi +C\delta$$

where:

(14) $$\begin{array}{c} A_{\gamma }(t)=\left[\begin{array}{cc} A_{\theta }(t) & A_{12}(t)\\ A_{21}(t) & A_{\varphi }(t) \end{array}\right]\\ A_{\theta }(t)=\left[\begin{array}{ccc} a_{22} & a_{23} & 0\\ a_{32} & a_{33} & a_{34}\\ 0 & \cos\gamma (t) & 0 \end{array}\right]\;\text{and}\;A_{\varphi }(t)=\left[\begin{array}{ccc} a_{22} & a_{23} & 0\\ a_{32} & a_{33} & 0\\ 0 & \cos\gamma (t) & 0 \end{array}\right] \end{array}$$

Nonzero off-diagonal coupling terms are contained in the system matrix **A***_γ_*(*t*)

(15) $$A_{12}(t)=-A_{21}(t)=\left[\begin{array}{ccc} \omega_{y}(t)v_{0}m_{x} & -\omega_{y}(t)(my_{cg}+Z_{\dot{q}}) & 0\\ -\omega_{y}(t)v_{0}(my_{cg}+Z_{\dot{q}}) & \omega_{y}(t)(J_{z}-J_{y}) & 0\\ 0 & -\sin\gamma (t) & 0 \end{array}\right]$$

where *v*_0_ is the initial velocity of glider at certain depth in the sea.

Equation (16) shows kinematic coupling among attitudes:

(16) $$\left[\begin{array}{c} \dot{\theta }\\ \dot{\varphi } \end{array}\right]=T_{3}\;\left[\begin{array}{cc} \cos\gamma (t) & -\sin\gamma (t)\\ \sin\gamma (t) & \cos\gamma (t) \end{array}\right]\;\left[\begin{array}{c} \omega_{x}\\ \omega_{z} \end{array}\right]$$

where T_3_ is the coefficient.

The non-diagonal terms are not zero because the roll angle *γ* is non-zero (when the roll is changing), therefore pitch rate *ω_x_* and heading rate *ω_z_* are no longer the time derivatives of pitch angle *θ* and heading angle *φ* for non-zero roll angle.

### 3.2. Backtracking Decoupling

According to the analysis above, the roll motion leads to the cross-coupling among attitude angles, which causes three orientation misalignment angles *ϕ_x_*, *ϕ_y_*, *ϕ_z_* erroneous. Attitudes correction equation is:

(17) $$C_{\text{nco}}^{b}=C^{T}C_{n}^{b}$$

where $C_{\mathrm{nco}}^{b}$ is the attitude matrix after correction; $C^{T}=\left[\begin{array}{ccc} 1 & \phi_{z} & -\phi_{y}\\ -\phi_{z} & 1 & \phi_{x}\\ \phi_{y} & -\phi_{x} & 1 \end{array}\right]$ is the attitude correction matrix; $C_{n}^{b}$ is the attitude matrix.

According to Equation (17), $C_{\mathrm{nco}}^{b}$ is erroneous because **C***^T^* is ill-conditioned. The quaternion equations are:

(18) $$\begin{array}{c} \left|q_{0}\right|=\frac{1}{2}\sqrt{1+C_{\text{nco}}^{b}(0,0)+C_{\text{nco}}^{b}(1,1)+C_{\text{nco}}^{b}(2,2)}\\ \left|q_{1}\right|=\frac{1}{2}\sqrt{1+C_{\text{nco}}^{b}(0,0)-C_{\text{nco}}^{b}(1,1)-C_{\text{nco}}^{b}(2,2)}\\ \left|q_{2}\right|=\frac{1}{2}\sqrt{1-C_{\text{nco}}^{b}(0,0)+C_{\text{nco}}^{b}(1,1)-C_{\text{nco}}^{b}(2,2)}\\ \left|q_{3}\right|=\frac{1}{2}\sqrt{1-C_{\text{nco}}^{b}(0,0)-C_{\text{nco}}^{b}(1,1)+C_{\text{nco}}^{b}(2,2)} \end{array}$$

$1+C_{\text{nco}}^{b}(0,0)+C_{\text{nco}}^{b}(1,1)+C_{\text{nco}}^{b}(2,2)$under the radical sign may become negative, which can cause the quaternion *q*_0_ erroneous. The same error also occurs for the quaternion *q*_1_, *q*_2_ and *q*_3_ in Equation (18):

(19) $$\begin{array}{c} \text{Heading}\;\varphi =\text{arctan}\left[\frac{C_{\text{nco}}^{b}(1,0)}{C_{\text{nco}}^{b}(1,1)}\right]\\ \text{Pitch}\;\theta =\text{arcsin}\left[C_{\text{nco}}^{b}(1,2)\right]\\ \text{Roll}\;\gamma =-\text{arctan}\left[\frac{C_{\text{nco}}^{b}(0,2)}{C_{\text{nco}}^{b}(2,2)}\right] \end{array}$$

are erroneous in the correction phase and the incorrect quaternion will lead to attitude solution wrong in the following update phase, and the attitude angle error is growing continuously in the later process. In addition, the error of attitude matrix also results in the specific force erroneous.

In order to reduce the errors caused by cross-coupling among attitude angles, the paper proposes the backtracking decoupling method. The procedure for implementing the backtracking decoupling can be summarized below.

For Equation (18), if the value under the radical sign occurs negative, namely:

$$\begin{array}{c} 1+C_{\text{nco}}^{b}(0,0)+C_{\text{nco}}^{b}(1,1)+C_{\text{nco}}^{b}(2,2)<0,1+C_{\text{nco}}^{b}(0,0)-C_{\text{nco}}^{b}(1,1)-C_{\text{nco}}^{b}(2,2)<0,\\ 1-C_{\text{nco}}^{b}(0,0)+C_{\text{nco}}^{b}(1,1)-C_{\text{nco}}^{b}(2,2)<0,1-C_{\text{nco}}^{b}(0,0)-C_{\text{nco}}^{b}(1,1)+C_{\text{nco}}^{b}(2,2)<0, \end{array}$$

use the last quaternions to reverse the attitude matrix. The specific steps are as follows:

Step 1: calculate $\omega_{\text{nb}}^{b}=\omega_{\text{ib}}^{b}-C_{n}^{b}(\omega_{\text{ie}}^{n}+\omega_{\text{en}}^{n})$, where $\omega_{nb}^{b}$ represents the angular velocity of the rotation projections of the carrier coordinate relative to the navigation coordinate frame on the carrier coordinate; $\omega_{ib}^{b}$ represents the angular velocity of the rotation projections of the carrier coordinate relative to the inertial coordinate frame on the carrier coordinate.
Step 2: calculate $\Delta \Theta =\int_{t_{k}}^{t_{k+1}}M^{*}(\omega_{\text{nb}}^{b})dt$ with $\omega_{\text{nb}}^{b}$ derived from Step 1, where:
  $$M^{*}(\omega_{\text{nb}}^{b})=[\begin{array}{cccc} 0 & -\omega_{\text{nb}}^{\text{bx}} & -\omega_{\text{nb}}^{by} & -\omega_{\text{nb}}^{bz}\\ \omega_{\text{nb}}^{\text{bx}} & 0 & \omega_{\text{nb}}^{bz} & -\omega_{\text{nb}}^{by}\\ \omega_{\text{nb}}^{by} & -\omega_{\text{nb}}^{bz} & 0 & \omega_{\text{nb}}^{\text{bx}}\\ \omega_{\text{nb}}^{bz} & \omega_{\text{nb}}^{by} & -\omega_{\text{nb}}^{\text{bx}} & 0 \end{array}]\;;$$
Step 3: calculate new quaternion $Q(t_{k+1})=\left[I\text{cos}\frac{\Delta \theta }{2}+\Delta \Theta \frac{\text{sin}\frac{\Delta \theta }{2}}{\Delta \theta }\right]Q(t_{k})$;
Step 4: normalize the quaternion by dividing the element $\sqrt{q_{0}+q_{1}+q_{2}+q_{3}}$;
Step 5: compute the new matrix to get the accurate attitude matrix;
Step 6: compute the attitudes according to Equation (19) and the calculate the specific force with the attitude matrix derived from Step 5;

Through the method above, the cross-coupling among attitude angles is eliminated effectively.

## 4. Adaptive EKF Based on Quaternion Expanded to the State Variable

### 4.1. The Quaternion Expanded to the State Variable

The state equation and the measurement equation of the first-order linear continuous system are respectively:

(20) $$\begin{array}{c} \dot{X}(t)=F(t)X(t)+G(t)W(t)\\ Z(t)=H(t)X(t)+V(t) \end{array}$$

The continuous system is described with differential equations in the practical application, so the continuous system needs to be discretized as follows:

(21) $$X_{k}=\Phi_{k,k-1}X_{k-1}+\Gamma_{k-1}W_{k-1}$$

(22) $$Z_{k}=H_{k}X_{k}+V_{k}$$

where **X***_k_* is the state vector at the moment of *k*; **Z***_k_* is observation vector at the moment of *k*; **Φ***_k_*,*_k_*_−1_ is the state transition matrix from the time of *k* −1 to *k*; Based on matrix **F**, state transition matrix **Φ** is calculated as: $\Phi_{k}=I+F\Delta t+\frac{1}{2}F^{2}{\left(\Delta t\right)}^{2}$, Δ*t* is a sampling interval; matrices **F** and **G** can be established by error equations; **Γ***_k_*_−1_ is the system noise matrix at the moment of *k* −1; **W***_k_*_−1_ is system noise;**H***_k_* is the observation matrix at the moment of *k* and **V***_k_* is observation noise. Commonly, **W***_k_*_−1_ and **V***_k_* are white noise sequences with zero mean; the variance matrix are **Q** and **R**, respectively.

The quaternion is expanded to the state vector given by:

(23) $$X={\left[\begin{array}{llllll} \begin{array}{ccc} \delta L & \delta \lambda & \delta h \end{array} & \begin{array}{ccc} \delta V_{E} & \delta V_{N} & \delta V_{U} \end{array} & \begin{array}{ccc} \varphi_{E} & \varphi_{N} & \varphi_{U} \end{array} & \begin{array}{ccc} \nabla_{bx} & \nabla_{by} & \nabla_{bz} \end{array} & \begin{array}{ccc} \varepsilon_{bx} & \varepsilon_{by} & \varepsilon_{bz} \end{array} & \begin{array}{cccc} \delta q_{0} & \delta q_{1} & \delta q_{2} & \delta q_{3} \end{array} \end{array}\right]}^{T}$$

The real velocity of the vehicle is $\left(\begin{array}{ccc} V_{E}^{R} & V_{N}^{R} & V_{U}^{R} \end{array}\right)$ in the East-North-Upward (ENU) axes, the velocity measured by the INS is:

(24) $$\{\begin{array}{c} V_{E}^{\text{INS}}=V_{E}^{R}+\delta V_{E}^{\text{INS}}\\ V_{N}^{\text{INS}}=V_{N}^{R}+\delta V_{N}^{\text{INS}}\\ V_{U}^{\text{INS}}=V_{U}^{R}+\delta V_{U}^{\text{INS}} \end{array}$$

where $\delta V_{E}^{\text{INS}}$, $\delta V_{N}^{\text{INS}}$, $\delta V_{U}^{\text{INS}}$ are the measurement errors of INS along the east, north and upward directions, respectively.

The velocity estimated by DR is:

(25) $$\{\begin{array}{c} V_{E}^{\text{DR}}=V_{E}^{R}+\delta V_{E}^{\text{DR}}\\ V_{N}^{\text{DR}}=V_{N}^{R}+\delta V_{N}^{\text{DR}}\\ V_{U}^{\text{DR}}=V_{U}^{R}+\delta V_{U}^{\text{DR}} \end{array}$$

where $\delta V_{E}^{\text{DR}}$, $\delta V_{N}^{\text{DR}}$, $\delta V_{U}^{\text{DR}}$ are the estimated errors by DR along the east, north and upward directions, respectively.

The real attitude angle of the vehicle is $\left(\begin{array}{ccc} \varphi^{R} & \theta^{R} & \gamma^{R} \end{array}\right)$ in the ENU axes, and heading angle, pitch angle and roll angle measured by the gyroscope are respectively:

(26) $$\{\begin{array}{c} \varphi^{\text{Gyro}}=\varphi^{R}+\delta \varphi^{\text{Gyro}}\\ \theta^{\text{Gyro}}=\theta^{R}+\delta \theta^{\text{Gyro}}\\ \gamma^{\text{Gyro}}=\gamma^{R}+\delta \gamma^{\text{Gyro}} \end{array}$$

where *δφ^Gyro^*, *δθ^Gyro^*, *δγ^Gyro^* are the corresponding attitude error estimates by gyroscopes along the east, north and upward directions, respectively.

Heading angle measured by the magnetometer is:

(27) $$\varphi^{\text{Mag}}=\varphi^{R}+\delta \varphi^{\text{Mag}}$$

Pitch angle and roll angle measured by the accelerometer are respectively:

(28) $$\{\begin{array}{c} \theta^{\text{Acce}}=\theta^{R}+\delta \theta^{\text{Acce}}\\ \gamma^{\text{Acce}}=\gamma^{R}+\delta \gamma^{\text{Acce}} \end{array}$$

where *δθ^Acce^*, *δγ^Acce^* are the corresponding attitude error estimates by accelerometers along the east, north and upward directions, respectively.

The observation vector **Z***_k_* is:

(29) $$Z_{k}=\left[\begin{array}{c} \begin{array}{c} V_{E}^{\text{INS}}-V_{E}^{\text{DR}}\\ V_{N}^{\text{INS}}-V_{N}^{\text{DR}}\\ V_{U}^{\text{INS}}-V_{U}^{\text{DR}} \end{array}\\ \begin{array}{c} \varphi^{\text{Gyaro}}-\varphi^{\text{Mag}}\\ \theta^{\text{Gyaro}}-\theta^{\text{Acce}}\\ \gamma^{\text{Gyaro}}-\gamma^{\text{Mag}} \end{array} \end{array}\right]=\left[\begin{array}{c} \begin{array}{c} \delta V_{E}^{\text{INS}}-\delta V_{E}^{\text{DR}}\\ \delta V_{N}^{\text{INS}}-\delta V_{N}^{\text{DR}}\\ \delta V_{U}^{\text{INS}}-\delta V_{U}^{\text{DR}} \end{array}\\ \begin{array}{c} \delta \varphi^{\text{Gyaro}}-\delta \varphi^{\text{Mag}}\\ \delta \theta^{\text{Gyaro}}-\delta \theta^{\text{Acce}}\\ \delta \gamma^{\text{Gyaro}}-\delta \gamma^{\text{Mag}} \end{array} \end{array}\right]=\left[\begin{array}{c} H_{V}\\ H_{\text{Att}} \end{array}\right]X_{k}+\left[\begin{array}{c} V_{V}\\ V_{\text{Att}} \end{array}\right]$$

where **H***_v_* is the velocity observation noise; **H***_Att_* is the attitude observation noise; **V***_V_* is the velocity measurement noise matrix; **V***_Att_* is the attitude measurement noise matrix.

### 4.2. Adaptive EKF

Define the measurement model as Equation (30) and assume two types of measurement noises [23].

(30) $$Z_{k}=H_{k}X_{k}+V_{k}=H_{k}X_{k}+V_{k}^{1}+\tau V_{k}^{2}$$

where $V_{k}^{1}$ is the measurement noise with a fixed variance, *τ* is a real number, *τ* ≥ 0, and $V_{k}^{2}$ is the time-varying measurement noise. Assume that the two types of measurements noises are uncorrelated.

Equation (31) shows the measurement residue considering the measurement value and the estimated value of the state variables:

(31) $$\delta Z_{k}=H_{k}X_{k}+V_{k}-H_{k}{\hat{X}}_{k/k-1}+V_{k}^{1}+\tau V_{k}^{2}=H_{k}(X_{k}-{\hat{X}}_{k/k-1})+V_{k}^{1}+\tau V_{k}^{2}+\xi (X_{k},{\hat{X}}_{k/k-1})$$

where *ξ*(**X***_k_*,**X̂***_k_*_/_*_k_*_−1_) is the higher order term in the estimation error. Equation (31) can be simplified to Equation (32) if this element is neglected. The ignored value can be considered as measurement noise.

(32) $$\delta Z_{k}=H_{k}{\tilde{X}}_{k/k-1}+V_{k}^{1}+\tau V_{k}^{2}$$

where **X̃***_k_*_/_*_k_*_−1_ = **X***_k_*− **X̂***_k_*_/_*_k_*_−1_ is the estimation error. The variance of the residual from Equation (32) is expressed as:

(33) $$S_{k/k-1}=E\left\{\delta Z_{k}\delta Z_{k}^{T}\right\}=H_{k}P_{k/k-1}H_{k}^{T}+R_{k}^{1}+\mu R_{k}^{2}$$

where *μ* = *τ*^2^, $R_{k}^{1}=E\left\{V_{k}^{1}{\left(V_{k}^{1}\right)}^{T}\right\}$, and $R_{k}^{2}=E\left\{V_{k}^{2}{\left(V_{k}^{2}\right)}^{T}\right\}$:

(34) $$P_{k/k-1}=\Phi_{k/k-1}P_{k-1/k-1}\Phi_{k/k-1}^{T}+Q_{d}$$

Substitute Equation (34) into Equation (33), thus:

(35) $$S_{k/k-1}=H_{k}P_{k/k-1}H_{k}^{T}+R_{k}^{1}+\mu R_{k}^{2}=H_{k}(\Phi_{k/k-1}P_{k-1/k-1}\Phi_{k/k-1}^{T}+Q_{d})H_{k}^{T}+R_{k}^{1}+\mu R_{k}^{2}=Y_{1}+\mu Y_{2}$$

where $Y_{1}=H_{k}P_{k/k-1}H_{k}^{T}+R_{k}^{1}=H_{k}(\Phi_{k/k-1}P_{k-1/k-1}\Phi_{k/k-1}^{T}+Q_{d})H_{k}^{T}+R_{k}^{1}$ and $Y_{2}=R_{k}^{2}$.

The measurement residual information is contained in Equation (35). It is possible to calculate the mean of the variance expressed as Equation (36) using the *N* residual from Equation (31):

(36) $$C=\frac{1}{N}\sum_{i=0}^{N}\left(\delta Z_{k-N+i}\delta Z_{k-N+i}^{T}\right)$$

The *μ* value of the adaptive filter can be obtained from Equations (35) and (36) as both of equations simultaneously contain the residual information. According to the Frobenius norm, the cost equation is defined as Equation (37) to calculate *μ* in real time. Then the *μ* value to minimize the Frobenius norm is obtained below:

(37) $$\underset{\mu \geq 0}{\text{min}}\left\{\Pi (\mu )={\left\|C-Y_{1}-\mu Y_{2}\right\|}^{2}\right\}$$

where ‖•‖^2^ is the Frobenius norm and ‖**Λ**‖^2^ = *tr*(**ΛΛ***^T^*). Rewrite Equation (37) as follows:

(38) $$\begin{array}{l} \Pi (\mu )={\left\|C-Y_{1}-\mu Y_{2}\right\|}^{2}=tr\left[(C-Y_{1}-\mu Y_{2}){\left(C-Y_{1}-\mu Y_{2}\right)}^{T}\right]\\ =\mu^{2}tr(Y_{2}Y_{2}^{T})-2\mu tr\left[\left(C-Y_{1})Y_{2}^{T}\right)\right]+tr{\left[\left(C-Y_{1})(C-Y_{1}\right)\right]}^{T} \end{array}$$

Minimize Π(*μ*) by $\frac{\partial \Pi (\mu )}{\partial \mu }=0$ to derive *μ* value. Take the derivative of Equation (38) and then obtain Equation (39):

(39) $$\frac{\partial \Pi (\mu )}{\partial \mu }=2\mu tr(Y_{2}Y_{2}^{T})-2tr\left[\left(C-Y_{1})Y_{2}^{T}\right)\right]$$

(40) $$\hat{\mu }=\frac{tr\left[\left(C-Y_{1})Y_{2}^{T}\right)\right]}{tr(Y_{2}Y_{2}^{T})}$$

The estimated value *μ̂* is substituted into Equation (35) during the filtering procedure.

The proposed adaptive EKF is summarized below:

(41) $${\hat{X}}_{k/k-1}={\hat{X}}_{k-1}+\int_{t_{k-1}}^{t_{k}}f({\hat{X}}_{t/t_{k}})dt$$

(42) $$P_{k/k-1}=\Phi_{k/k-1}P_{k-1}\Phi_{k/k-1}^{T}+Q_{d}$$

(43) $$Y_{1}=H_{k}P_{k/k-1}H_{k}^{T}+R_{k}^{1}=H_{k}(\Phi_{k/k-1}P_{k-1}\Phi_{k/k-1}^{T}+Q_{d})H_{k}^{T}+R_{k}^{1}$$

(44) $$Y_{2}=R_{k}^{2}$$

(45) $$C=\frac{1}{N}\sum_{i=0}^{N}\left(\delta Z_{k-N+i}\delta Z_{k-N+i}^{T}\right)$$

(46) $$\hat{\mu }=\frac{tr\left[\left(C-Y_{1})Y_{2}^{T}\right)\right]}{tr(Y_{2}Y_{2}^{T})}$$

(47) $$S_{k/k-1}=H_{k}P_{k/k-1}H_{k}^{T}+R_{k}^{1}+\hat{\mu }R_{k}^{2}$$

(48) $$K_{k}=P_{k/k-1}H_{k}^{T}S_{k/k-1}^{-1}$$

(49) $${\hat{X}}_{k}={\hat{X}}_{k/k-1}+K_{k}(Z_{k}-H_{k}{\hat{X}}_{k/k-1})$$

(50) $$P_{k}=P_{k/k-1}-K_{k}H_{k}P_{k/k-1}$$

## 5. Simulation Results and Analysis

**Figure 2.**
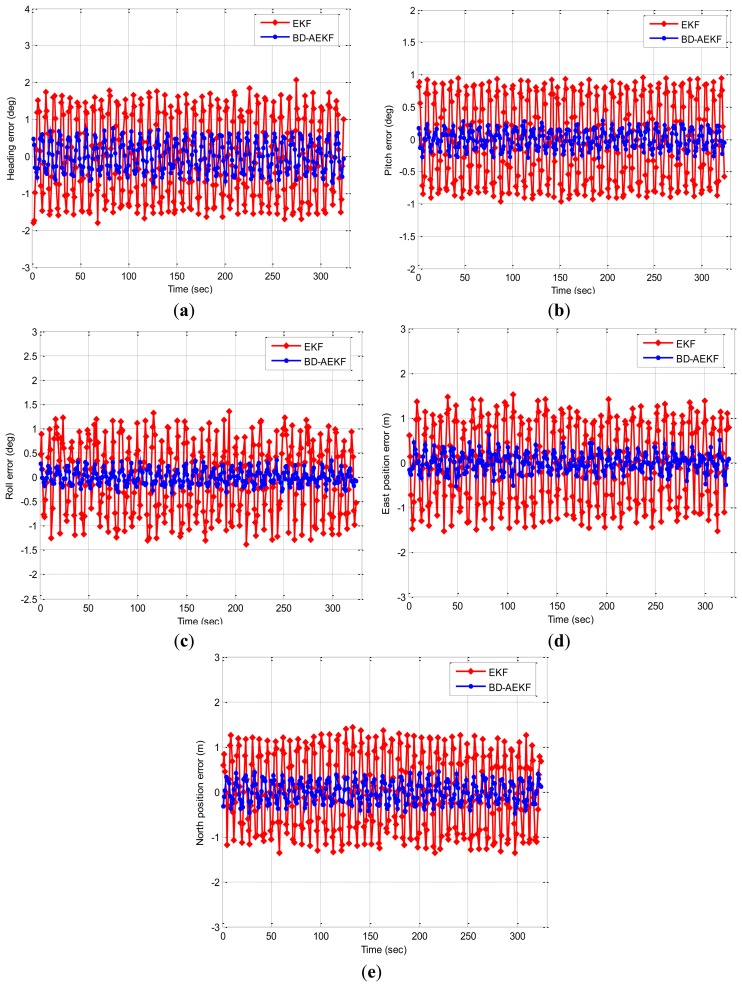
The attitude error and position error for EKF and BD-AEKF when the pitch changes in the sine form. (**a**) Heading (**b**) Pitch (**c**) Roll (**d**) East position (**e**) North position.

The pitch motion and roll motion of an underwater glider are simulated to evaluate the performance of the BD-AEKF method proposed in this paper. The simulations include: (1) the first simulation is that the pitch angle changes in the sine form (*θ* = 30sin*t*; *θ* is the pitch angle and *t* is the time) while keeping the heading and roll unchanged; (2) the second simulation is that the roll angle changes in the sine form (*γ* = 30sin*t*; *γ* is the roll angle and *t* is the time) while keeping the heading and pitch unchanged. These simulations are done to demonstrate whether the BD-AEKF method can eliminate cross-coupling, smooth the filtering output and improve the accuracy. The simulation initial conditions are as follows: the simulation time is 330 s; the sample frequency is 1 Hz; the initial heading angle is 45°; the initial pitch angle and roll angle are 0°; the angular rate bias is 0.02 °/s (RMS); the angular rate random walk is $6{}^{\circ}/\sqrt{hr}$; the linear acceleration bias is 0.3 mg (RMS); the linear acceleration random walk is $0.06m/s/\sqrt{hr}$; the linear velocities in three directions are all zero. The first simulation results are shown in Figure 2. In Figure 2, the attitude angles errors for EKF and BD-AEKF are shown in Figure 2a–c, respectively. The position errors for EKF and BD-AEKF in east direction and north direction are shown in Figure 2d,e, respectively. Moreover, the root mean square errors (RMSE) of attitude and position for EKF, BD-AEKF are shown in Table 1.

From Figure 2 it can be clearly seen that the performance of BD-AEKF is much better than EKF. When the pitch is changing in the sine form, the cross-coupling among three attitude angles becomes more serious, and it can cause the attitude and position calculation to be inaccurate or even erroneous. The errors cannot be reduced for EKF, however the BD-AEKF method can eliminate the cross-coupling and further smooth the filtering output after decoupling. Therefore, the accuracy and stability of the attitude and position determination are greatly improved.

In Table 1, the heading RMSE for BD-AEKF is 0.4164°, which is lower than the EKF. Because of the cross-coupling, the heading angle vibrates periodically while the pitch is changing in the sine form. The cross-coupling can be eliminated by BD-AEKF so the errors can be reduced correspondingly, as seen from Figure 2a. In Figure 2b the cross-coupling affects the pitch accuracy, and the RMSE of pitch for EKF is 0.6432°, however the RMSE for BD-AEKF is 0.1442°. Comparing Figure 2c with Figure 2a, like for the heading, the roll is affected by the cross-coupling when the pitch is swaying, but the effect on the roll due to cross-coupling is not greater than the effect on the heading due to cross-coupling, which it is proved in Table 1. Moreover, for the EKF the oscillation of the heading is more regular than the oscillation of the roll when the pitch is swaying. By employing the BD-AEKF, the oscillation of heading and the oscillation of roll are all avoided effectively. In Figure 2d, the theoretical east position is zero, but it oscillates regularly when the pitch is swaying for the EKF. The RMSE in east direction for BD-AEKF is 0.2119 m which is reduced greatly compared with the RMSE for EKF. In Figure 2e, the error of the north position for BD-AEKF is reduced and the RMSE decreases from 0.8400 m to 0.2331 m.

**Figure 3.**
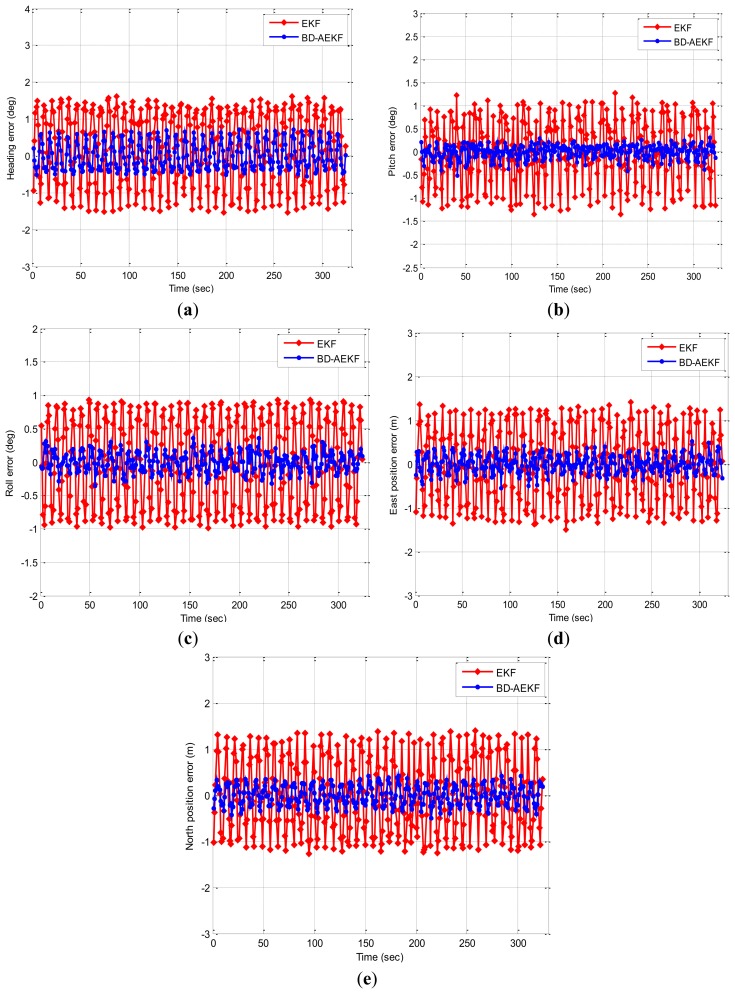
The attitude error and position error for EKF and BD-AEKF when the roll changes in the sine form. (**a**) Heading (**b**) Pitch (**c**) Roll (**d**) East position (**e**) North position.

The second simulation results are shown in Figure 3. In Figure 3, the attitude errors for EKF and BD-AEKF are shown in Figure 3a–c, respectively. The position errors for EKF and BD-AEKF in east direction and north direction are shown in Figure 3d,e, respectively. RMSE of attitude and position for EKF, BD-AEKF are shown in Table 2.

Like in Figure 2, it is seen from Figure 3 that the errors of attitude and position for BD-AEKF are smaller than the errors of attitude and position for EKF. In Table 2, the RMSE of heading for BD-AEKF is reduced from 1.0069° to 0.3751°. For the pitch error, the improvement in RMSE for BD-AEKF is reduced from 0.6812° to 0.1453°. In Figure 3c, the BD-AEKF also has the lower roll error. The RMSE for the BD-AEKF is 0.1466°, however, the RMSE for the EKF is 0.6488°. In Figure 3d,e, the theoretical east position and north positions are zero, but they vibrate periodically for EKF when the roll is changing in the sine form. By employing the BD-AEKF, the RMSE in the east position for BD-AEKF is reduced from 0.8297 m to 0.2050 m and the RMSE of the north position is reduced from 0.8104 m to 0.2194 m.

## 6. Experiments and Results

In order to assess the performance of the proposed BD-AEKF algorithm, a new inertial system is designed in our lab (Model number: SUNS-2). This system consists of a Digital Signal Processing (DSP) and Inertial Measurement Unit (IMU). The characteristics of the SUNS-2 are shown in Table 3.

### 6.1. The Experiment Based on a Three-Axis Non-Magnetic Turntable

In this experiment, a three-axis non-magnetic turntable is employed to validate whether the cross-coupling among attitudes is eliminated by using the proposed backtracking decoupling method when pitch or roll are changing. The specifications for this turntable are shown in Table 4.

#### 6.1.1. Experiment When Backing Decoupling Is Not Used

Adjust the inner frame, the middle frame and the external frame of the turntable to local level with initialization zero point. Then SUNS-2 is fixed on the center of the turntable. Rotate the pitch axis of the turntable every 10° while keeping the heading and roll axes of the turntable unchanged, and the data is sampled by a Personal Computer (PC). The sample time is approximately 1 min in every sampling location and the range of pitch axis rotation is from −60° to +60°. Record the attitude angle outputs and the results are shown in Table 5.

After that, return the turntable to the initial location and rotate the roll axis of the turntable every 10° while keeping other two axes of the turntable unchanged. The range of roll axis rotation is from −60° to +60°. The attitude angle outputs results are shown in Table 6.

It is clearly seen from Table 5 that the heading changes greatly with the pitch motion. The heading change is theoretically 0° when the pitch axis is rotating. However, the mean heading change is 2.7362°, and the standard deviation of the heading is 9.9466°, and the heading change maximum is 3.8.85°. Similarly, the roll also changes along with pitch motion. The roll change is theoretically 0°. However, the mean roll change is 1.4487°, and the standard deviation of roll is 4.3701°, and the maximum deviation of roll change is 2.3092°.

The results for roll motion are similar to those for pitch motion above as one can observe clearly in Table 6. Thus, it is concluded that the performance of attitude determination is very poor when the pitch axis or roll axis is rotating due to the cross-coupling among attitude angles. Large attitude errors can be introduced and the conventional method is no longer effective in many practical underwater glider applications.

#### 6.1.2. The Experiment When Backing Decoupling Is Used

In order to solve the cross-decoupling problem, the backing decoupling method is proposed. The same experiment procedures as Section 6.1.1 are done to validate the performance of proposed method. Rotate the pitch and roll axes of turntable from −60° to +60°, respectively. The attitude angle outputs results are shown in Tables 7 and 8, respectively.

In Table 7, by employing the backing decoupling, the heading changes little along with pitch motion and the heading change is close to the theoretical value of 0°. The mean of heading change is 0.4422° now, while the mean of heading change was 2.7362° before using the proposed backing decoupling. The standard deviation of heading is 0.5434° which is much lower than before. The heading change maximum is reduced from3.8085° before to 0.5992° now.

Moreover, it is easily to see that the roll changes little along with pitch motion in Table 7. The mean of roll change, the standard deviation of roll and roll change maximum are 0.2066°, 0.2940°, 0.3999°, respectively, which are all lower than before using the proposed method.

Compared with Table 7, similar results are obtained from Table 8. The heading change and pitch change are close to the theoretical value of 0° when the roll is changing. The mean of heading change, the standard deviation of heading, and heading change maximum are 0.2949°, 0.5275°, 0.5858°, respectively. The mean of pitch change, the standard deviation of pitch, and pitch change maximum are 0.2397°, 0.2164°, 0.3992°, respectively.

Therefore, it is concluded that the performance of attitude determination is improved because the cross-coupling among three attitude angles is eliminated by proposed backing decoupling.

### 6.2. The Vehicle Experiments

**Figure 4.**
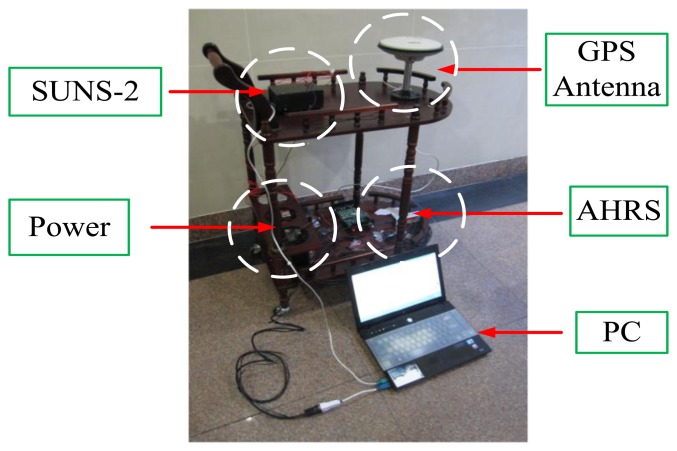
The vehicle experiment platform.

After eliminating cross-decoupling, to evaluate whether the proposed BD-AEKF can further smooth the filtering output to improve the accuracy and stability of attitude and position determination, real vehicle experiments were done in the playground of Southeast University. The vehicle with equipment is shown in Figure 4. The trajectories of the experiments are a straight line and a rectangle, respectively. The velocity of the vehicle is about 0.5 m/s.

The Attitude and Heading Reference System (AHRS) is used as the attitude reference and the specifications for AHRS are shown in Table 9. The GPS receiver (JAVAD GNSS) is used as the position reference and the performance of the GPS receiver is given in Table 10. The sample time used in this work is 1 s.

#### 6.2.1. The Vehicle Experiment in Straight Line Trajectory

**Figure 5.**
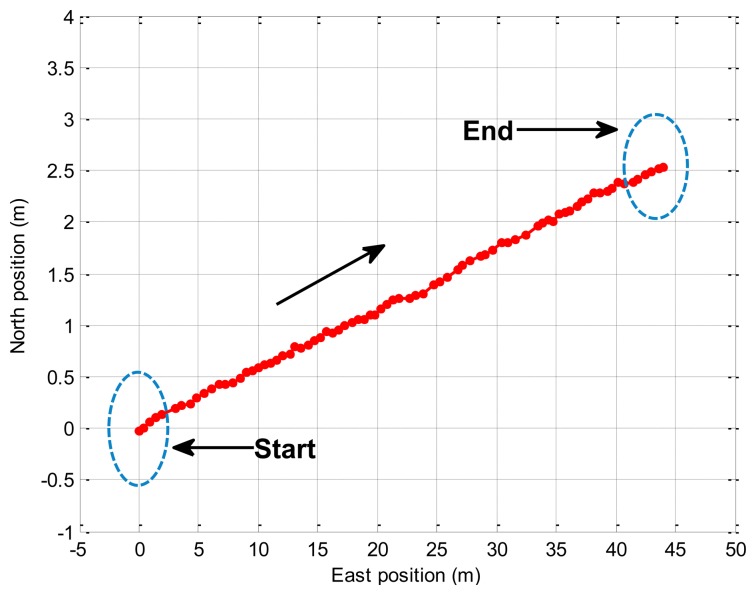
The trajectory of the real experiment (Line).

**Figure 6.**
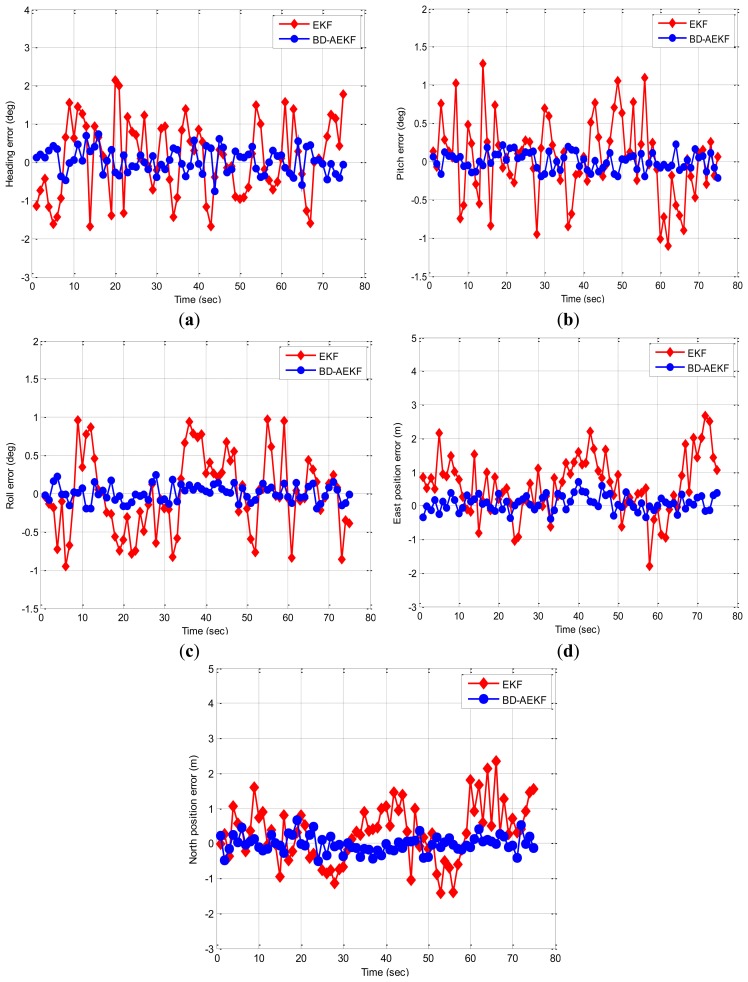
The errors for EKF and BD-AEKF in the line trajectory. (**a**) Heading (**b**) Pitch (**c**) Roll (**d**) East position (**e**) North position.

In this experiment, the trolley runs along the straight line (about 40 m) in the playground, and Figure 5 displays the trajectory of the real experiment. The trolley runs from the start point to the end point. In Figure 6, the attitude errors for the EKF and the proposed BD-AEKF are shown in Figure 6a–c, respectively. The position errors in the east direction and north direction for the EKF and the BD-AEKF are shown in Figure 6d,e, respectively. Moreover, the RMSE of attitude and position for the EKF and BD-AEKF are shown in Table 11.

As shown in Figure 6, it can be seen that the performance of the BD-AEKF is better than EKF in terms of attitude and position estimation. During the trolley running process, the pitch and roll are vibrating continuously in a small range because of the practical road implementation, so the cross-coupling is obvious. The BD-AEKF eliminates the cross-coupling and smoothes the filtering output, therefore the errors of attitude and position determination are greatly reduced, however, the traditional EKF cannot solve the cross-coupling problem among three-attitude and the errors are relatively larger. In Figure 6a, the heading error for EKF is much larger than the heading error for BD-AEKF. In Table 11, the RMSE of heading for BD-AEKF is 0.3278°, which is lower than the EKF. Similarly, the pitch and roll errors are reduced by the BD-AEKF. The RMSE of pitch is reduced from 0.5142° to 0.1140° and the RMSE of roll is reduced from 0.5043° to 0.1090°. From Figure 6d,e, it can be seen that the estimation accuracy in terms of east position and north position for BD-AEKF is superior to that for EKF. The BD-AEKF reduces the RMSE of east position from 0.9547 m to 0.2486 m compared with EKF. The RMSE of north position for BD-AEKF is 0.2629 m, which is lower than the RESE for EKF.

#### 6.2.2. The Vehicle Experiment in the Rectangle Trajectory

**Figure 7.**
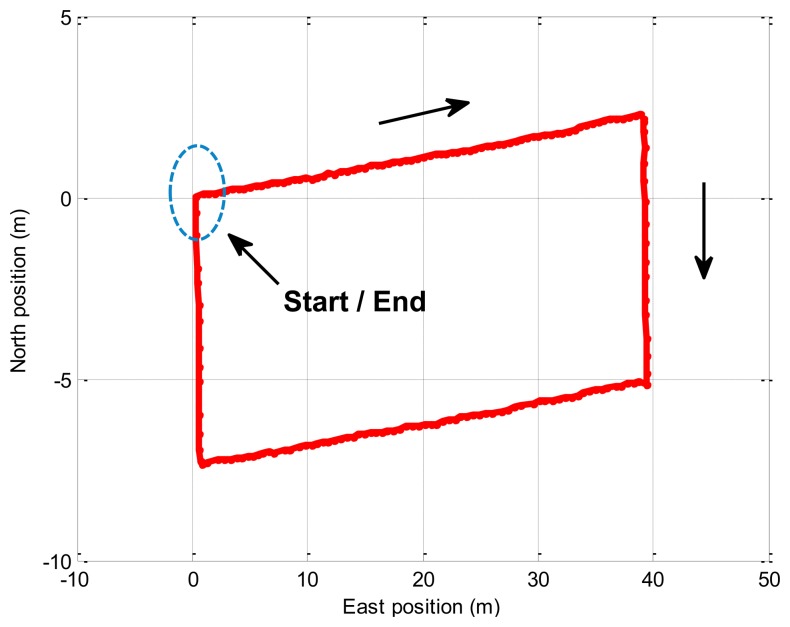
The trajectory of the real experiment (rectangle).

**Figure 8.**
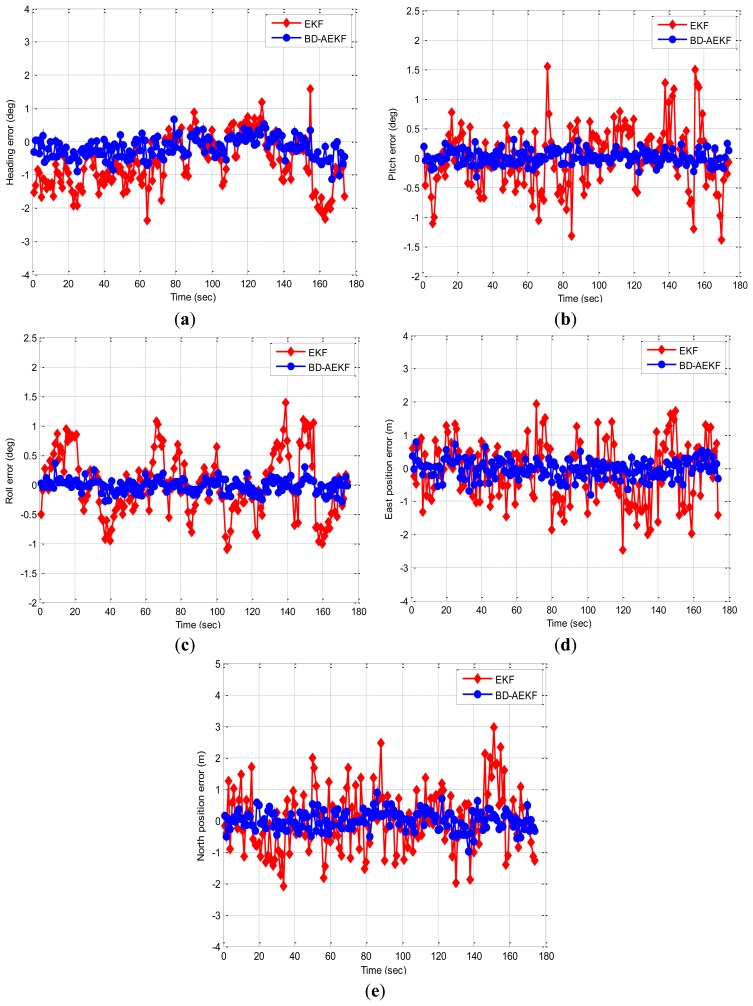
The errors for EKF and BD-AEKF in the rectangle trajectory. (**a**) Heading (**b**) Pitch (**c**) Roll (**d**) East position (**e**) North position.

In this experiment, the trolley runs along the rectangle trajectory (length about 40 m; width about 8 m). Figure 7 displays the trajectory of the real experiment. Figure 8 shows error comparison results between methods. The attitude errors for the EKF and the BD-AEKF are shown in Figure 8a–c, respectively. The position errors for the EKF and the BD-AEKF in the east direction and north direction are shown in Figure 8d,e, respectively. Furthermore, the RMSE of attitude and position for the EKF and BD-AEKF are shown in Table 12.

Figure 8 shows that the BD-AEKF performs better than the EKF in the attitude and position determination. In Figure 8a, the BD-AEKF is able to reduce the heading error and it decreases the RMSE of the heading from 0.9651° to 0.3422° compared with the EKF in Table 12. Similarly, it is easy to see from Figure 8b,c that the BD-AEKF is effective at reducing the pitch error and the roll error, respectively. The RMSE of the pitch for the BD-AEKF is 0.1201° which is lower than that for EKF. The RMSE of the roll is reduced from 0.5374° to 0.1135°. For the position error, the BD-AEKF also has lower error than the EKF from Figure 8d,e. The RMSE of the east position is 0.2797 m for the BD-AEKF while the RMSE is 1.0198 m for the EKF. The BD-AEKF reduces the RMSE of the north position from 0.9970 m to 0.2946 m.

As can be seen from the simulations (Section 5) and the practical vehicle experiments (Section 6), it is concluded that the traditional EKF may be not suitable for a practical underwater glider. EKF uses linearized models by applying first order approximation to nonlinear systems. However, when the non-linearity is severe, EKF often gives unreliable or divergent estimates. The pitch and roll motion are common for underwater gliders. When pitch or roll motion appears, the cross-coupling among the three attitude angles becomes more obvious, which can cause the estimation of attitude and position to be inaccurate or even erroneous, therefore the EKF method cannot match the system model of the underwater glider well. In order to overcome EKF shortcomings, the BD-AEKF is proposed to solve this problem.

## 7. Conclusions

In order to eliminate the cross-coupling between attitudes and to improve the accuracy of attitude and position determination, this paper proposes the BD-AEKF method. The backtracking decoupling can eliminate effectively the cross-coupling among the three attitude angles when pitch or roll motion occurs. As the basis of decoupling, the AEKF based on quaternion expanded to the state variable further smoothes the filtering output. An improvement is achieved for the accuracy and stability of attitude and position determination. A new underwater navigation system is designed. Three-axis non-magnetic turntable experiments and vehicle experiments are done to assess the performance of BD-AEKF. The experimental results show the BD-AEKF method is more effective in terms of decoupling and navigation accuracy improvement than the traditional method in practical glider applications.

## Figures and Tables

**Table 1.** Comparison of errors between EKF and BD-AEKF (*θ* = 30sin*t*).

	**RMSE**	

	**EKF**	**BD-AEKF**
Heading (degree)	1.1426	0.4164
Pitch (degree)	0.6432	0.1442
Roll (degree)	0.7422	0.1505
East position (m)	0.8771	0.2119
North position (m)	0.8400	0.2331

**Table 2.** Comparison of errors for EKF and BD-AEKF (*γ*=30sin*t*).

	**RMSE**	

	**EKF**	**BD-AEKF**
Heading (degree)	1.0069	0.3751
Pitch (degree)	0.6812	0.1453
Roll (degree)	0.6488	0.1466
East position (m)	0.8297	0.2050
North position (m)	0.8104	0.2194

**Table 3.** The physical characteristics for the SUNS-2 used in this work.

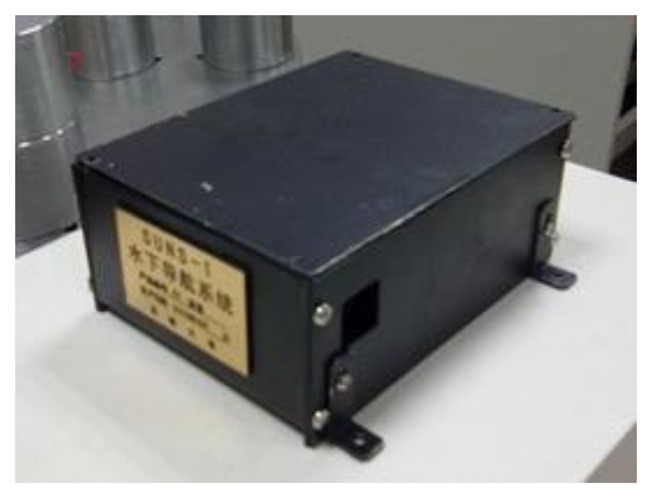	**Physical Characteristics**	

	Length (m)	0.11
	Width (m)	0.07
	Height (m)	0.05
	Volume (dm^3^)	0.385
	Weight (g)	<250
	Power (w)	<0.6

**Table 4.** The specifications for the three-axis non-magnetic turntable.

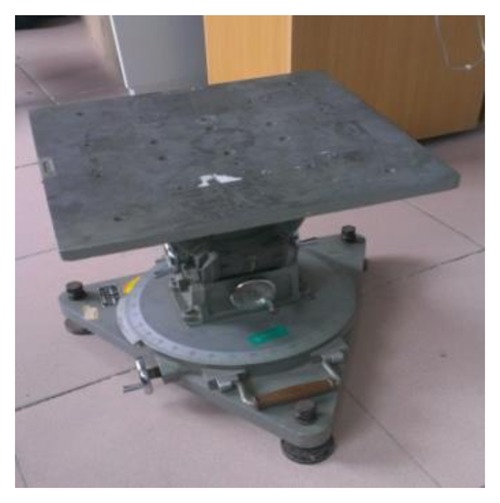	**Attitude**	**Accuracy (Degree)**	**Range (Degree)**

	Heading	0.05	0 to 360
	Pitch	0.1	−90 to 90
	Roll	0.1	−90 to 90

**Table 5.** The outputs of heading and roll while pitch is changing (not using backing decoupling).

**Pitch (Degree)**	**Heading (Degree)**	**Change of Heading (Degree)**	**Roll (Degree)**	**Change of Roll (Degree)**
−60	51.0849	---	−0.8305	---
−50	54.6034	3.5185	1.4786	2.3091
−40	57.6925	3.0891	3.3713	1.8927
−30	60.3745	2.6820	5.4105	2.0392
−20	63.0753	2.7008	7.3860	1.9755
−10	65.1080	2.0327	6.8183	−0.5677
0	66.9587	1.8507	8.1123	1.2940
10	69.0162	2.0575	8.7630	0.6507
20	71.3348	2.3186	9.9801	1.2171
30	74.3130	2.9782	9.5737	−0.4064
40	76.8575	2.5445	11.0065	1.4328
50	80.1108	3.2533	12.6449	1.6384
60	83.9193	3.8085	14.6059	1.9610

**Table 6.** The outputs of heading and pitch while roll is changing (not using backing decoupling).

**Roll (Degree)**	**Heading (Degree)**	**Change of Heading (Degree)**	**Pitch (Degree)**	**Change of Pitch (Degree)**
−60	117.7436	---	1.1355	---
−50	114.6767	−3.0669	−0.6905	−1.8260
−40	111.5195	−3.1572	−2.3900	−1.6995
−30	109.3491	−2.1704	−3.8728	−1.4828
−20	107.2472	−2.1019	−4.9485	−1.0757
−10	106.8687	−0.3785	−4.5106	0.4379
0	105.1737	−1.6950	−5.7121	−1.2015
10	103.1754	−1.9983	−6.7665	−1.0544
20	101.3348	−1.8406	−6.5154	0.2511
30	99.1213	−2.2135	−7.3497	−0.8343
40	98.5010	−0.6203	−8.7032	−1.3535
50	97.0836	−1.4174	−10.3746	−1.6714
60	94.4144	−2.6692	−12.2939	−1.9193

**Table 7.** The outputs of heading and roll while pitch is changing (using backing decoupling).

**Pitch (Degree)**	**Heading (Degree)**	**Change of Heading (Degree)**	**Roll (Degree)**	**Change of Roll (Degree)**
−60	−19.4526	---	0.9587	---
−50	−19.9302	−0.4776	0.4054	−0.3533
−40	−19.0930	0.5372	0.8053	0.3999
−30	−18.1468	0.5462	0.6411	−0.1642
−20	−18.7931	−0.5463	0.4798	−0.1613
−10	-18.6250	0.1681	0.7728	0.2930
0	−18.3728	0.2522	0.8253	0.0525
10	−19.1769	−0.5041	0.7961	−0.0292
20	−19.2889	−0.1120	0.6954	−0.1007
30	−19.4729	−0.5840	0.2007	−0.3947
40	-19.0737	0.5992	0.1198	−0.0809
50	−19.4991	−0.4254	0.2024	0.0826
60	−18.2455	0.5536	0.9695	0.3671

**Table 8.** The outputs of heading and pitch while roll is changing (using backing decoupling).

**Roll (Degree)**	**Heading (Degree)**	**Change of Heading (Degree)**	**Pitch (Degree)**	**Change of Pitch (Degree)**
−60	−17.6152	---	−0.3280	---
−50	−17.5019	0.1133	−0.1888	0.1392
−40	−16.6161	0.5858	−0.4880	−0.3992
−30	−17.5656	0.0505	−0.7251	0.2629
−20	−17.5411	−0.5755	−0.4539	0.2712
−10	−17.8651	−0.3240	−0.1247	0.3292
0	−17.7535	0.1116	−0.4090	−0.3843
10	−17.6658	0.0877	−0.6390	0.1700
20	−17.3516	0.3142	−0.4314	0.2076
30	−17.7248	−0.3732	−0.1096	0.3218
40	−18.2573	−0.5325	−0.2018	−0.0922
50	−18.4609	−0.2036	−0.0251	0.1767
60	−18.7272	−0.2663	−0.1467	−0.1216

**Table 9.** The specifications for the attitude reference system (AHRS).

**Attitude**	**Performance**		
	Range	degree	0 to 360
Heading	Static Accuracy at Normal Conditions	degree RMS	0.2
	Static Accuracy in Temperature Range	degree RMS	0.5
	Dynamic Accuracy	degree RMS	0.7
Pitch, Roll	Range	degree	−90 to +90,
			−180 to +180
	Static Accuracy at Normal Conditions	degree RMS	0.04
	Static Accuracy in Temperature Range	degree RMS	0.1
	Dynamic Accuracy	degree RMS	0.4
Physical characteristics	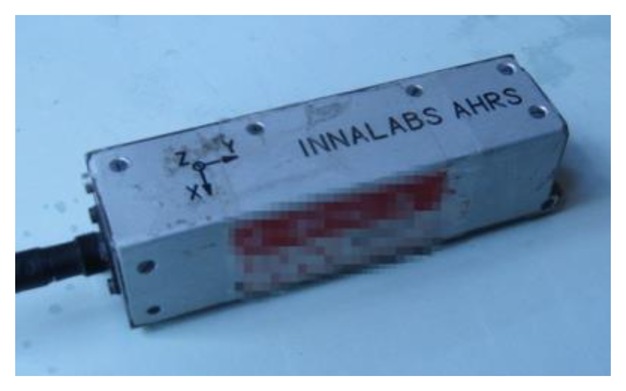		

**Table 10.** The specifications for the position reference system (GPS).

	**Autonomous Accuracy**	**<2 m**
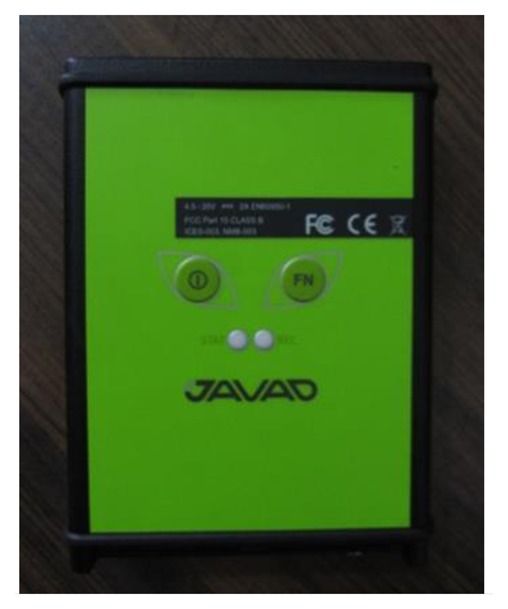	**Static, Fast Static Accuracy**	Horizontal: 0.3 cm + 0.5 ppm*base_line_length Vertical: 0.5 cm + 0.5 ppm*base_line_length
	**Kinematic Accuracy**	Horizontal: 1 cm + 1 ppm*base_line_length Vertical: 1.5 cm + 1.5 ppm*base_line_length
	**RTK (OTF) Accuracy**	Horizontal: 1 cm + 1 ppm*base_line_length Vertical: 1.5 cm + 1.5 ppm*base_line_length
	**DGPS Accuracy**	< 0.25 m Post Processing < 0.5 m Real Time

**Table 11.** Comparison of errors between EKF and BD-AEKF (Line).

	**RMSE**	

	**EKF**	**BD-AEKF**
Heading (degree)	0.9015	0.3278
Pitch (degree)	0.5142	0.1140
Roll (degree)	0.5043	0.1090
East position (m)	0.9547	0.2486
North position (m)	0.9277	0.2629

**Table 12.** Comparison of errors between EKF and BD-AEKF (Rectangle).

	**RMSE**	

	**EKF**	**BD-AEKF**
Heading (degree)	0.9651	0.3422
Pitch (degree)	0.5295	0.1201
Roll (degree)	0.5374	0.1135
East position (m)	1.0198	0.2797
North position (m)	0.9970	0.2946
